# Unveiling the emergence of community-acquired methicillin-resistant *Staphylococcus aureus* (CA-MRSA) in Chinese hospitals with remarkable genetic diversity: a multicenter molecular epidemiological investigation

**DOI:** 10.1128/spectrum.03395-24

**Published:** 2025-10-30

**Authors:** Shangyu Tu, Xiaoyang Zhang, Qiaoli Xu, Wenyan Han, Yihua Cai, Si Liu, Jiaao Wang, Hui Wang, Hongbin Chen

**Affiliations:** 1 Department of Clinical Laboratory, Peking University People’s Hospital, Beijing, China; 2 Zhangzhou Affiliated Hospital of Fujian Medical University, Zhangzhou, Fujian, China; 3 Clinical Laboratory of the Second Affiliated Hospital of Inner Mongolia Medical University, Hohhot, Inner Mongolia, China; 4 Medical Laboratory of the Third Affiliated Hospital of Shenzhen University, Shenzhen, Guangdong, China; Icahn School of Medicine at Mount Sinai, New York, New York, USA

**Keywords:** community-acquired methicillin-resistant *Staphylococcus aureus *(CA-MRSA), whole-genome sequencing, phylogeny, ST59

## Abstract

To understand the prevalence and phylogeny of methicillin-resistant *Staphylococcus aureus* (MRSA) in Chinese hospitals and identify the dominant MRSA lineages causing hospital infections, we collected 101 *S*. *aureus* isolates from multiple centers across China, tested their antibiotic sensitivity, and performed whole-genome sequencing. Using our group’s database, a core genome-based phylogenetic analysis of 204 *S*. *aureus* isolates was conducted. The results revealed an interspersed distribution of MRSA and MSSA without distinct clade separation. CC59-ST59-t437-SCC*mec* IV was identified as the predominant community-associated MRSA (CA-MRSA) clone widely distributed across cities, while hospital-associated MRSA (HA-MRSA) was mainly composed of CC5-ST5-SCC*mec* II and CC8-ST239-SCC*mec* III, forming significant phylogenetic clusters. In contrast, MSSA strains were more diverse, with no clear clustering patterns. No regional clustering was observed, suggesting a widespread dissemination of major clones nationwide. Notably, some sequence types (e.g., ST45 and ST59) were shared by both MRSA and MSSA, and closely related strains with and without SCC*mec* elements suggested that smaller SCC*mec* types may be more readily gained or lost during evolution. Our findings indicate that CA-MRSA has a significant presence in hospitals with diverse subtypes, highlighting the need for increased attention to CA-MRSA in S. *aureus* control and prevention.

**IMPORTANCE:**

The aim of this study was to identify the molecular epidemiological characteristics of *S. aureus* in Chinese hospitals, especially the dominant typing changes of MRSA during transmission. This study found that CA-MRSA appeared in the hospital environment with high diversity, suggesting that clinical attention should be paid to CA-MRSA in the prevention and diagnosis of *S. aureus* infection.

## INTRODUCTION

Methicillin-resistant *Staphylococcus aureus* (MRSA) is one of the most important pathogens causing nosocomial infections (1). Due to its high pathogenicity and resistance to multiple antibiotics, the control of MRSA remains a major challenge. Based on the source of acquisition, MRSA can often be divided into community-associated MRSA (CA-MRSA) and hospital-associated MRSA (HA-MRSA). The two are different in terms of molecular typing, virulence, and genetic elements carried (2, 3).

Since its detection, MRSA has emerged into different clones, and clones were detected worldwide. The most frequently reported clones belong to one of the five major clonal complexes (CCs), including CC5, CC8, CC22, CC30, and CC45 (4, 5). These CCs contain various STs and are distributed in many different countries or regions of the world. For example, CC5-ST5 are the most frequently reported CCs in Asian countries (6–8). While CC8-ST8 is common in the United States, CC22 is a globally widespread CC (9–11).

The MRSA of different lineages generally has the characteristics of regional prevalence (12). However, owing to factors, such as the widespread global dissemination of bacterial strains and the inappropriate use of antibiotics, previously dominant lineages in specific regions have been increasingly outcompeted—and, in some cases, entirely replaced—by strains belonging to other lineages (12).

Previous studies have confirmed that ST5 and ST239 were two types of MRSA prevalent in Chinese hospitals, but ST239 has a trend of being gradually replaced by CA-MRSA ST59 (13–15). In our previous research, we observed a significant decrease in the prevalence of HA-MRSA dominated by ST239, dropping from 42.4% in 2013 to 11.6% in 2016 (13). In conclusion, the gradual influx of CA-MRSA into hospitals in recent years is an epidemiological phenomenon that deserves attention (16).

Here, we tested the antibiotic susceptibility of 101 strains of *S. aureus* from 11 hospitals in eight cities in China during 2022, conducted a whole-genome sequencing for all strains, and used molecular typing and phylogenetic analyses to discuss their genetic characteristics.

## MATERIALS AND METHODS

### *S. aureus* isolates

**Fig 1:**
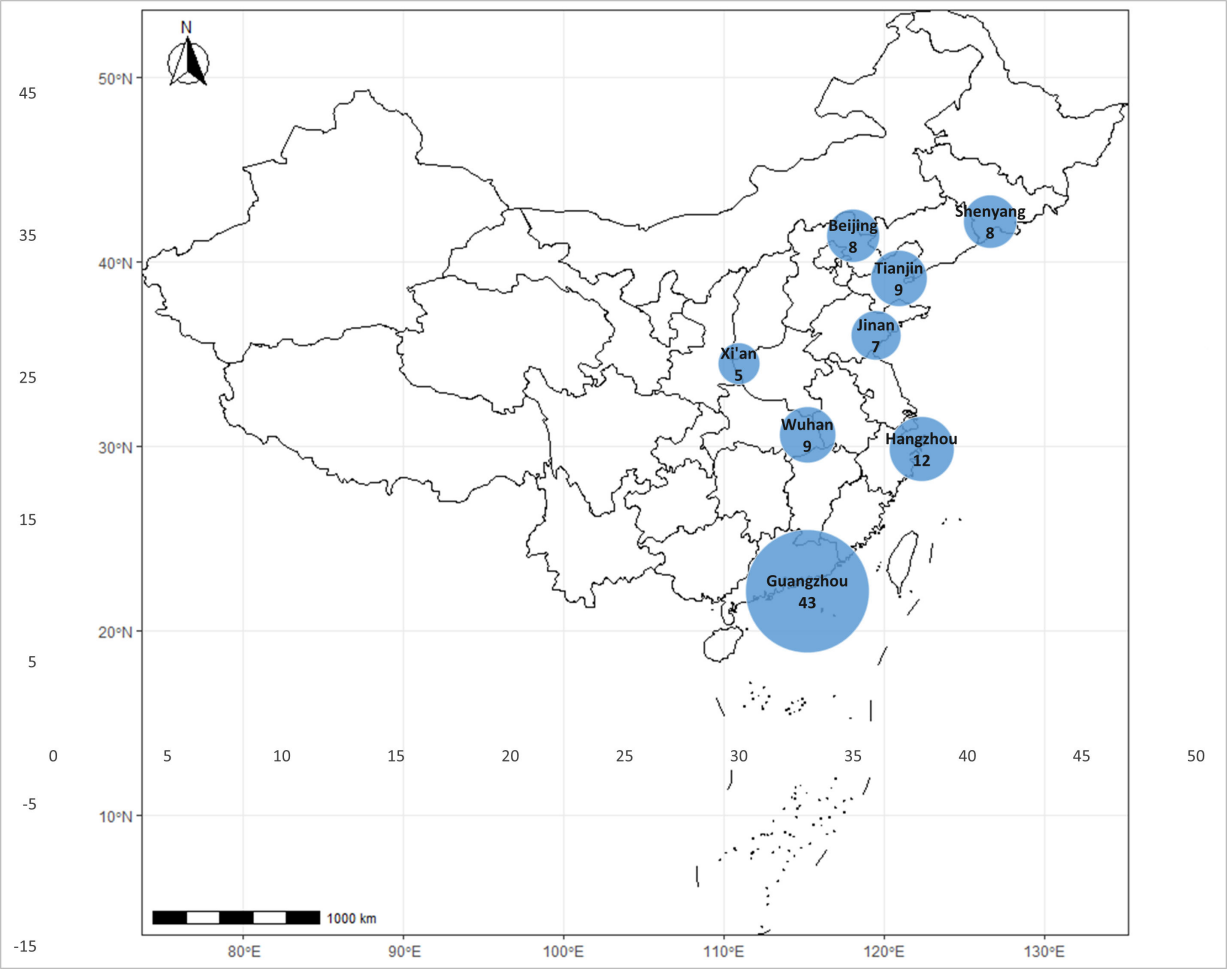
Geographical distribution of 101 *S*. *aureus* clinical isolates in this study. The circle size represents the number of isolates.

For this study, 101 *S. aureus* clinical isolates were collected from eight cities in China during 2022, namely, Beijing, Tianjin, Shenyang, Jinan, Xi'an, Wuhan, Hangzhou, and Guangzhou (Fig. 1). The isolates came from 10 hospitals, that is, two in Beijing, two in Guangzhou, and one in each of the other cities. All isolates were isolated from the clinical patients with bloodstream infection (64.4%, 65/101), abdominal infection (12.9%, 13/101), or hospital-acquired pneumonia (22.8%, 23/101). These strains were all identified as infecting rather than colonizing based on the clinical data at the time of collection. All isolates were non-duplicate, first isolates continuously collected from patients during the surveillance period. In cases where the same strain was isolated from multiple anatomical sites of the same patient, only the earliest isolate was included for analysis. Specifically, bloodstream infection strains were isolated from patients who had one or more positive blood cultures obtained more than 48 h after hospital admission. Strains from hospital-acquired pneumonia isolated from patients who developed pneumonia 48 h after hospitalization. Strains were derived from intraperitoneal infections, isolated from purulent secretions from intraperitoneal surgery or diagnostic punctures, or derived from the culture of drainage fluid from surgical drainage tubes. The study was approved by the research ethics board at Peking University People’s Hospital (2024PHB319-001).

### Antimicrobial susceptibility testing

Antibiotic susceptibility was assessed based on the minimum inhibitory concentration (MIC) values using the agar dilution method with the following antibiotics (all from Sigma Chemical unless otherwise specified): penicillin, oxacillin, ceftaroline, erythromycin, moxifloxacin, levofloxacin, ciprofloxacin, clindamycin, minocycline, rifampin, sulfamethoxazole, chloramphenicol, teicoplanin, vancomycin, tedizolid, and linezolid. The MICs of antibiotics were interpreted using Clinical and Laboratory Standards Institute breakpoints. All susceptibility data and molecular test results were analyzed using the software WHONET 23.14.20. Percentage values or frequencies were analyzed pairwise by two-tailed *χ* test or Fisher’s exact test.

### Whole-genome sequencing analysis

All of the *S. aureus* isolates were sequenced using Illumina technology with 150-base pair paired-end protocols on the NextSeq 500 System (Illumina). The average sequencing depth was 224.3 ± 57.92. An average of 4.34 ± 1.18 M read count was obtained from each MRSA isolate, and the guanine/cytosine (GC) rate was 32.72 ± 0.05. The contig number was 49.73 ± 59.57, with 217.9 ± 159.8 Kbp of N50. The *De novo* assembly of the genomes of all isolates was performed using SPAdes software (version 3.10.0) and annotated using Prokka software (version 1.12). All data underwent strict quality control; the effective percentage of the data was more than 99.5%; the sequencing error rate was stable at 0.03%; and the Q20 and Q30 values reached more than 96 and 90%, respectively. The GC content is 32.49–35.06%. Based on the genome size of 2.8 Mb, the coverage range is 470×–614×, which meets the depth requirements of the whole genome analysis. This Whole Genome Shotgun Project has been deposited at GenBank under accession number PRJNA1245266. The version described in this paper is version JBMQJJ000000000–JBMQNI000000000.

### Phylogenetic analysis

A total of 204 *Staphylococcus aureus* strains were included in this study for the phylogenetic analysis. Among them, 101 strains were newly generated sequencing data through whole-genome sequencing in this study, while the remaining 103 strains were sourced from the public database of our research group to complement the diversity of geographic distribution and molecular typing. The remaining 103 strains were derived from our in-house database (as described in [17, 18]) comprising previously sequenced isolates that fulfilled the following criteria: (i) isolated from China; (ii) obtained from bloodstream and abdominal infections or HAP; (iii) collected prior to 2022; and (iv) having complete molecular typing records. All downloaded data were re-annotated using the same bioinformatics pipeline to ensure consistency in typing standards. The core genome was extracted using Roary software (version 3.11.2), and its alignment was screened for recombination using ClonalFrameML software (version 1.2); putative recombinant regions were removed before the phylogenetic analysis. Maximum-likelihood phylogenetic trees were constructed using RAxML software (version 8.2.10) with a general time reversible model and 1,000 bootstrap replicates. For tree rooting, the chromosomal sequence of the *S. aureus* isolate MW2 (ST1) (NC_003923) was used as a closely related outgroup. The annotation was performed with iTOL (https://itol.embl.de/).

### Molecular typing analysis

The program MLST (version 2.0) in CGE (https://www.genomicepidemiology.org/services/) was utilized to perform MLST of the *S. aureus* assemblies. SCC*mec* and spa type of strains were determined using SCC*mec*Finder (version 1.2) and SpaTyper (version 1.0), respectively.

### Accessory genome analysis

The antibiotic resistance and virulence genes were identified by the program ResFinder (version 4.5.0) in CGE and annotated based on the Virulence Factors of Pathogenic Bacteria (VFDB) Database and the Comprehensive Antibiotic Resistance Database (CARD). The mobile genetic elements (MGEs) were determined using MGE (version 1.0.3) in CGE. The prophages were analyzed by PHASTER (https://phaster.ca/).

## RESULTS

### MRSA and MSSA had different antimicrobial susceptibility spectra

**Fig 2:**
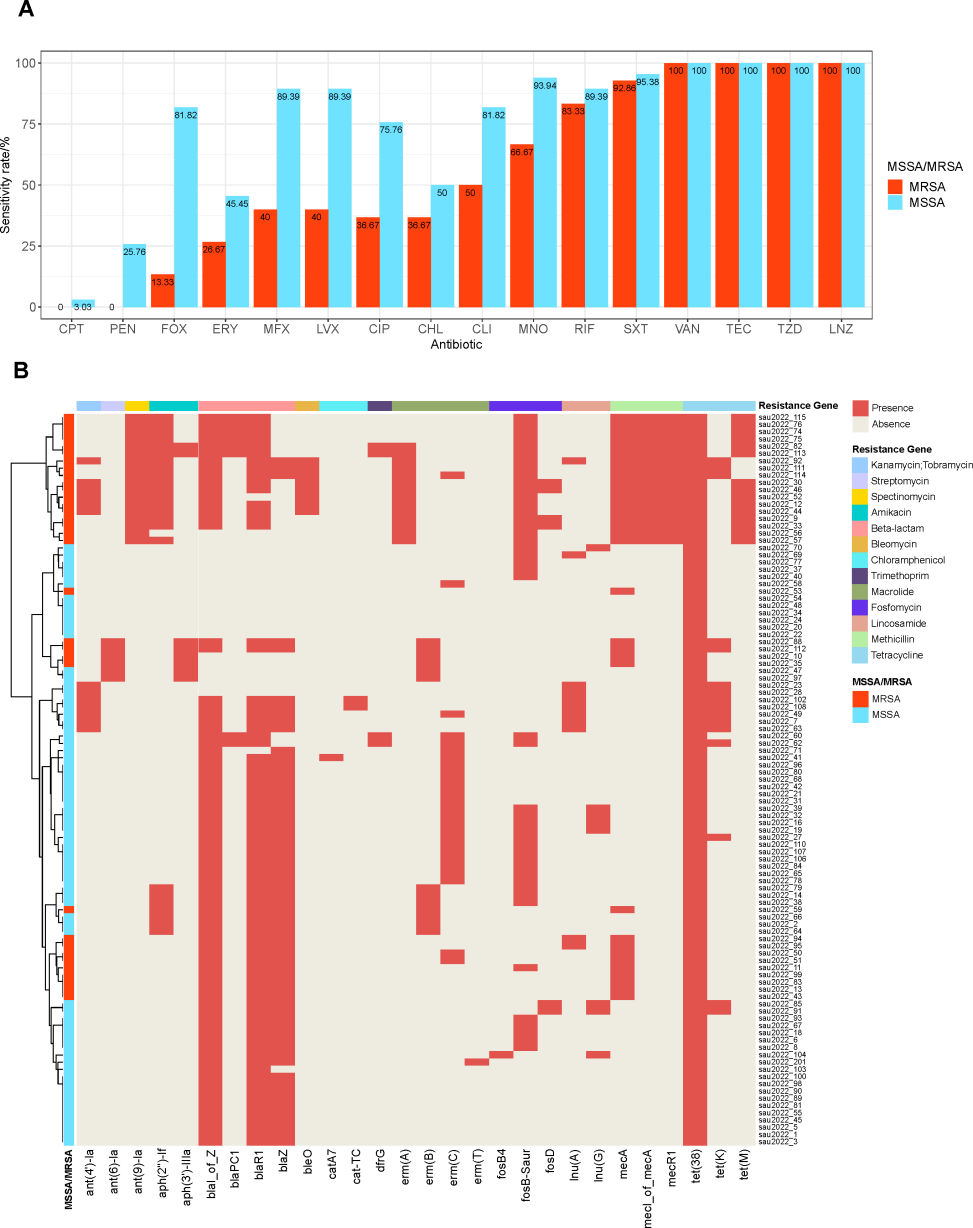
(**A**) Antimicrobial susceptibility of the *S. aureus* clinical isolates. CPT, ceftaroline; PEN, penicillin; FOX, cefoxitin; ERY, erythromycin; MFX, moxifloxacin; LVX, levofloxacin; CIP, ciprofloxacin; CHL, chloramphenicol; CLI, clindamycin; MNO, minocycline; RIF, rifampicin; SXT, sulfamethoxazole/trimethoprim; VAN, vancomycin; TEC, teicoplanin; TZD, tedizolid; and LNZ, linezolid. (**B**) Heat map of acquired resistance genes. The presence of this resistance gene in the heat map is shown in red. Resistance genes are divided into 13 groups according to the antibiotic they target, with each group distinguished by a different color, as shown in the legend.

Among all the *S. aureus* isolates, 33 were identified as MRSA, and 68 were identified as MSSA. The result of the antimicrobial susceptibility testing shows that MRSA and MSSA have different antimicrobial susceptibility spectra. In addition to β-lactam, MRSA had lower susceptibility to quinolones (moxifloxacin 42.11%, levofloxacin 42.11%, ciprofloxacin 39.47%), clindamycin (55.26%), and minocycline (73.68%) than MSSA (Fig. 2A). Rifampicin, cotrimoxazole, teicoplanin, vancomycin, taldizolid, and linezolid remain effective against the majority of MRSA with susceptibility rates of 94.29–97.39%. There was no significant difference in antimicrobial resistance among isolates of different infection types.

The acquired antimicrobial genes carried by MRSA and MSSA isolates are also different (Fig. 2B). The MRSA isolates had significantly higher carriage rates of *ant (9)-la* (54.5% vs. 0, *P* < 0.001), *aph(2'')-lf* (54.5% vs. 8.8%, *P* < 0.001), and *bleO* (24.2% vs. 0, *P* < 0.001) genes, which confer resistance to spectinomycin, amikacin, and bleomycin, respectively. Among macrolide resistance genes, *ermA* was more frequently detected in MRSA than in MSSA (42.4% vs. 0, *P* < 0.001), whereas *ermC* was more prevalent in MSSA (33.8% vs. 9.1%, *P* < 0.001). Although the MRSA isolates exhibited a markedly lower susceptibility rate to quinolones compared to MSSA (MFX:40% vs 89.39%, LVX:40% vs 89.39%, CIP:36.67 vs 50%), no acquired quinolone resistance genes were detected in either group, suggesting that MRSA resistance to quinolones is primarily due to chromosomal mutations in *grlA/B* and *gyrA*.

### Molecular typing characteristics of *S. aureus* isolates

Among 101 strains of *S. aureus*, 32 distinct sequence types (STs) and one unknown type were identified, among which ST188 (10.9%, 11/101), ST5 (9.9%, 10/101), ST59 (8.9%, 9/101), and ST764 (6.9%, 7/101) accounted for the most (Fig. S1B). These known STs belonged to 10 known clonal complexes (CCs), and the most common were CC5 (30.7%, 31/101), CC1 (15.8%, 16/101), CC59 (11.9%, 12/101), and CC8 (9.9%, 10/101) (Fig. S1A). The distribution of STs varied across different regions. For example, CC22-ST22 was the most prevalent type in Beijing, accounting for 50% (4/8) of the isolates. In Wuhan, CC1-ST188 was the dominant type, representing 33.3% (3/9). CC5-ST764 was detected exclusively in Guangzhou, where it constituted the largest proportion at 16.3% (7/43). The spa types of these isolates were diversified, including 44 known types and eight unknown types, among which t189 (9.9%, 10/101), t1084 (6.9%, 7/101), and t437 (6.9%, 7/101) were relatively large (Fig. S1C). In addition, the SCC*mec* analysis of MRSA showed that types IV (39.4%, 13/33) and II (36.4%, 12/33) were the main types, with a small amount of types III (15.2%, 5/33) and V (3.0%, 1/33) and unknown types (6.1%, 2/33) (Fig. S1D). The detailed molecular typing characteristics of all 101 isolates are provided (Table 1) .

**TABLE 1:** Molecular types of isolates^*b*^

CC types	MLST types^*a*^	Spa types^*a*^	MSSA/MRSA	SCCmec	Number of isolates
CC5	ST5	t548	MSSA	–	4
		t311	MRSA	II	3
		Others	MSSA	–	3
	ST764	t1084	MRSA	II	7
	ST6	t701	MSSA	–	4
		t304	MRSA	IV	1
	Others	Others	MSSA	–	7
		Others	MRSA	II	2
CC1	ST188	t189	MSSA	–	8
		t189	MRSA	IV	1
		Others	MSSA	–	2
	ST1	t127	MSSA	–	2
	Others	Others	MSSA	–	3
CC59	ST59	t437	MSSA	–	2
		t437	MRSA	IV	3
		t1151	MSSA	–	1
		t3523/t3527	MRSA	IV	3
	ST944/ST1640	Unknown	MSSA	–	2
	ST338	t437	MRSA	V	1
CC8	ST239	t459	MRSA	III	3
		t37	MRSA	III	2
		Unknown	MRSA	Unknown	1
	Others	Others	MSSA	–	4
CC22	ST22	t309	MSSA	–	4
		t223	MRSA	IV	2
CC7	ST7	Total	MSSA	–	5
CC15	ST15	Total	MSSA	–	5
CC398	ST398	t1250/t3625/Unknown	MSSA	–	4
		Unknown	MRSA	Unknown	1
CC45	ST45	t116	MSSA	–	1
			MRSA	IV	3
		t563	MSSA	–	1
CC2	ST88/ST965	Total	MSSA	–	4
Unknown	ST5926/Unknown	Total	MSSA	–	2

^*a*^ Table 1 only shows the main subtypes in each CC; "others" represents all the subtypes, except those specifically listed above; and "total" represents all the subtypes. The specific data can be found in Data set S1.

^*b*^ – indicates the isolates without an SCCmec element, which corresponding to MSSA strains.

### CA-MRSA emerged in hospitals in high diversity

A core genome-based phylogenetic tree was constructed using 204 *Staphylococcus aureus* isolates collected domestically, including 73 MRSA and 131 MSSA strains. The analysis integrated CC, MLST, and spa and SCC*mec* typing to comprehensively characterize their molecular epidemiology. The phylogeny revealed substantial genetic diversity among the isolates, with the MRSA and MSSA strains interspersed rather than forming distinct, separate clades (Fig. S2).

**Fig 3:**
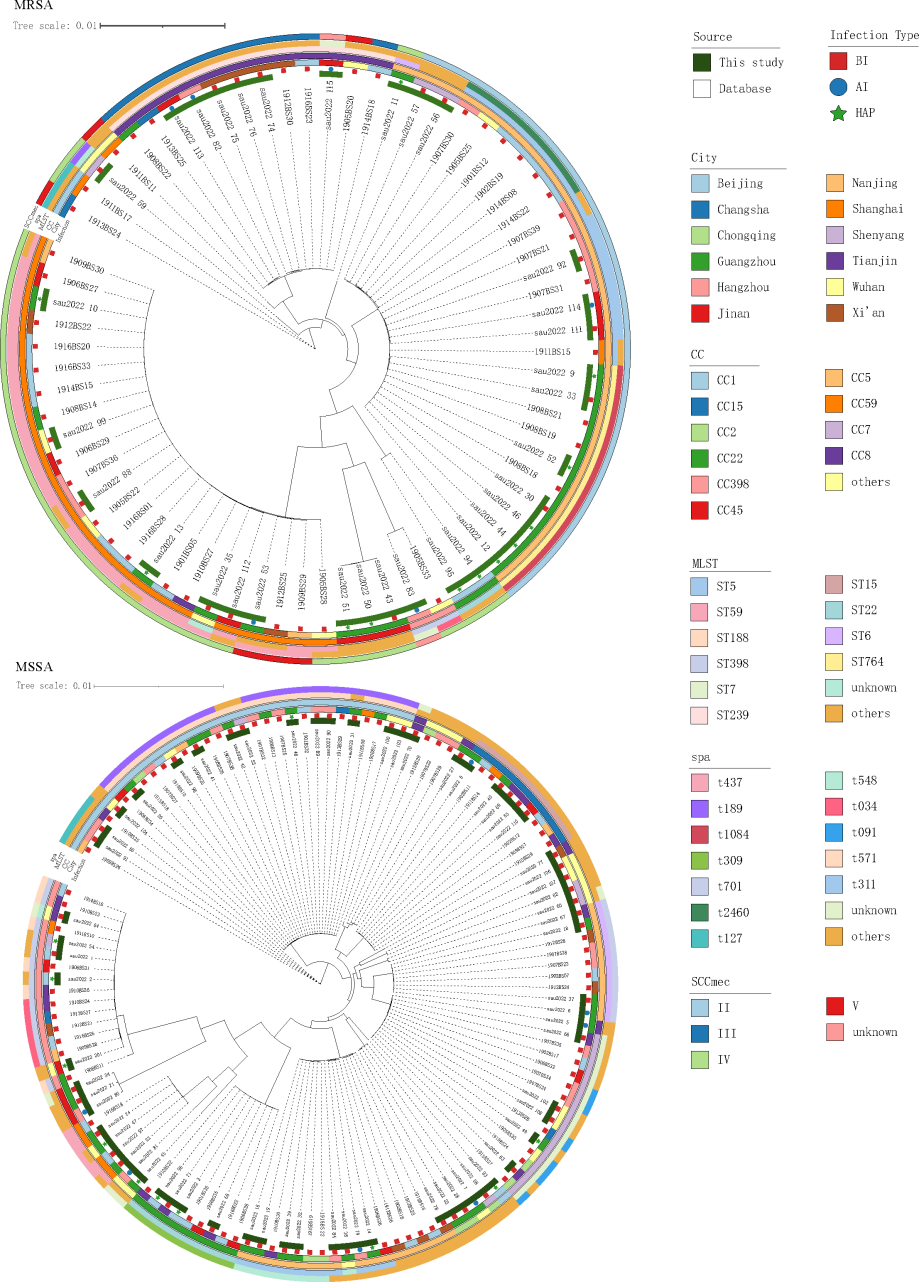
Core genome-based phylogenetic tree of *Staphylococcus aureus* isolates (*n* = 204). MRSA isolates are in the top half, while the MSSA isolates are in the bottom half. From the innermost to the outermost ring, the annotated metadata layers represent the infection type, city, CC, MLST, *spa* type, and SCC*mec* type, respectively. Metadata colors indicate different allele types or subtypes as defined in the legend.

Within the MRSA isolates, CC59-ST59 comprised approximately 30.1% (22/73) of the population and represented the predominant community-associated MRSA clone in China (Fig. 3). This clone predominantly harbored the SCC*mec* type IV (95.5%, 21/22) and was primarily associated with the spa type t437 (76.2%, 16/21). CC59-ST59 strains were widely distributed across all sampled cities, including Beijing, Guangzhou, Shanghai, and Wuhan, indicating extensive geographic dissemination. HA-MRSA was mainly composed of CC5-ST5, CC5-ST764, and CC8-ST239 lineages accounting for 37.1 (13/35), 28.6 (10/35), and 28.6% (10/35) of HA-MRSA isolates, respectively. Among HA-MRSA, all of the CC5 strains carried SCC*mec* type II (100%, 25/25), while all of the CC8 strains carried SCC*mec* type III (100%, 10/10). Both lineages formed statistically significant clusters on the phylogenetic tree, underscoring their roles as dominant hospital clones.

In contrast, MSSA was broadly distributed across multiple CCs, exhibiting a more heterogeneous distribution. Among these, CC1-ST188-t189 was the most prevalent (16.8%, 22/131), and molecular typing revealed considerable diversity without clear clustering patterns, reflecting a more complex and diverse epidemiological background for MSSA.

Importantly, isolates from different cities did not exhibit obvious regional clustering in the phylogeny; major clones were broadly distributed nationwide, suggesting that dominant *S. aureus* clones have transcended geographic boundaries and are widely disseminated across China.

Additionally, several STs, such as ST5, ST45, ST59, and ST398, were shared by both MRSA and MSSA strains. In ST45-t116 and ST59-t437 strains, both MRSA and MSSA isolates with identical typing profiles were identified, and they clustered on the same branch of the phylogenetic tree, indicating a close evolutionary relationship (Fig. S2). This phenomenon was observed exclusively in SCC*mec* type IV isolates, implying that smaller SCC*mec* elements may be more readily gained or lost during the evolution of *S. aureus*.

### Geographic and genetic correlation of *S. aureus* clones and resistance elements

**Fig 4:**
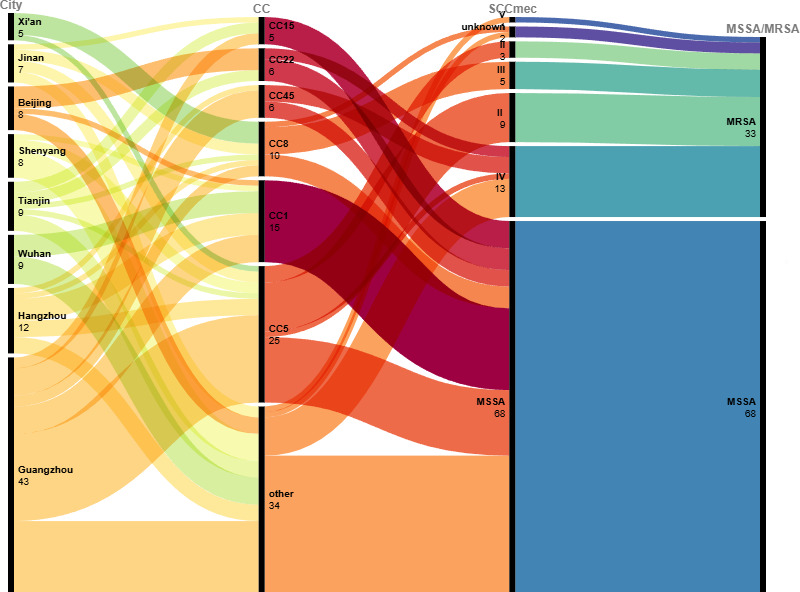
The Sankey map shows the relationship among the strain isolation region, clonal typing, and SCC*mec* type. Correlations between each group are represented with curved lines whose width is proportional to the number of isolates.

The Sankey map (Fig. 4) visually illustrates the correlation between regional distribution, CCs, and SCC*mec* type of *S. aureus*, highlighting the inter-city transmission dynamics. It clearly showed that *S. aureus* generally exhibits widespread inter-city transmission patterns. To further investigate potential region-specific clonal transmission patterns, we performed Fisher’s exact test on representative CCs from different cities. In Guangzhou, CC5 accounted for 44.2% (19/43) of isolates, significantly higher than in other regions (20.7%, 12/58, *P* = 0.016), indicating a local expansion of the CC5 lineage. Similarly, CC1 was more prevalent in Wuhan (44.4%, 4/9) compared to other cities (13.5%, 12/89, *P* = 0.033), suggesting a possible clonal dissemination of CC1 in this region. In Xi'an, CC8 constituted 80.0% (4/5) of isolates, markedly higher than in other areas (6.3%, 6/96; *P* < 0.01) and supporting the presence of a highly localized clonal expansion of CC8. Notably, detailed typing of CC1 isolates revealed that both Wuhan and Hangzhou shared the ST188-t189 subtype, suggesting potential interregional transmission. However, Hangzhou also harbored ST1-t127 strains, reflecting greater local diversity. These findings demonstrate that, although *S. aureus* exhibits widespread inter-city transmission, certain CC types may show regional dominance, likely reflecting localized outbreaks or sustained clonal propagation.

To further explore the regional variation in *S. aureus* clonal transmission and antimicrobial resistance, we also analyzed the distribution of SCC*mec* types across different CCs. Among 31 CC5 isolates, 38.7% (12/31) carried SCC*mec* type II, and 3.2% (1/31) carried SCC*mec* type V, while the remaining were MSSA strains. This suggests that CC5 is predominantly associated with MRSA and has a strong linkage to SCC*mec* II. Similarly, CC8 showed a substantial proportion of MRSA strains primarily carrying SCC*mec* III (50%, 5/10). In contrast, CC1 was mostly composed of MSSA strains (93.8%, 15/16), with only one isolate harboring SCC*mec* IV, implying a non-resistant clonal background. Notably, CC59 demonstrated a strong association with SCC*mec* IV (50%, 6/12) and included five MSSA and one SCC*mec* V strains, consistent with typical CA-MRSA features. These findings, when combined with our previous geographic analysis, suggest that certain resistant clones, such as CC5-SCC*mec* II in Guangzhou and CC8-SCC*mec* III in Xi'an, may have undergone local expansion, whereas the CC1-MSSA cluster in Wuhan likely reflects a non-resistant local transmission. Overall, these results highlight the potential for the region-specific clonal spread of both resistant and susceptible *S. aureus* lineages across China.

### The same clade carries a relatively specific MGE spectrum

**Fig 5:**
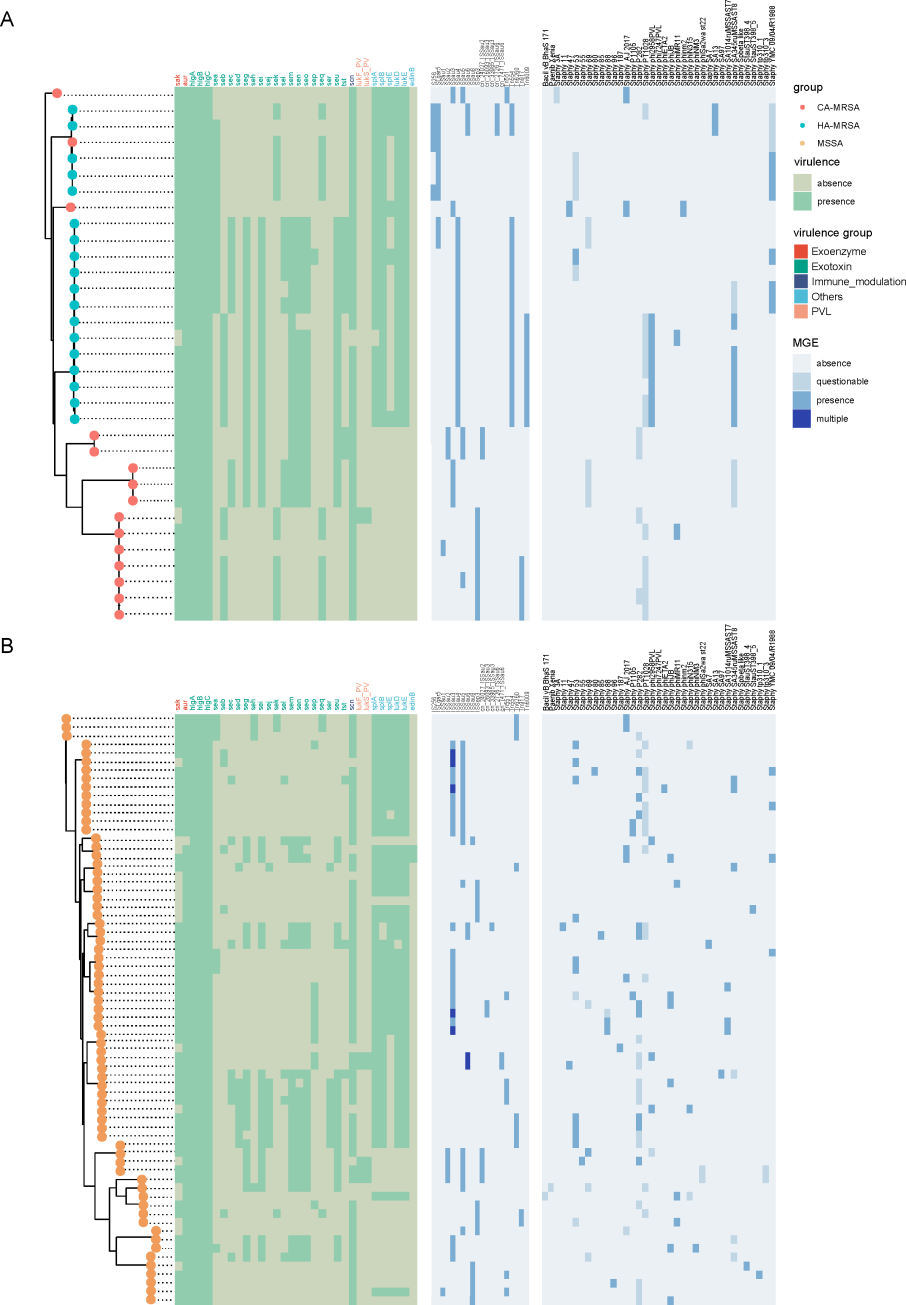
Presence/absence profiles of accessory genomes among Chinese MRSA (**A**) and MSSA (**B**) isolates. Maximum-likelihood phylogenetic trees of MRSA (**A**) and MSSA (**B**) isolates were constructed based on core genome alignments. Tree tips are color-coded according to SCC*mec* types, as indicated in the legend. The heatmaps illustrate the presence or absence of virulence genes and MGEs, including insertion sequences, transposons, and prophages. Virulence genes are shown in green, with dark green indicating presence and light green indicating absence. MGEs are displayed in blue, with increasing intensity representing absence (light blue), questionable presence (medium blue), confirmed presence (dark blue), and presence of multiple copies (deepest blue).

In total, MRSA had more virulence genes than MSSA (Fig. 5). Notably, 93.9% (31/33) of MRSA and 75.0% (51/68) of MSSA strains were positive for the *sak* gene, which encodes staphylokinase. The difference in the carriage rate between MRSA and MSSA was statistically significant (Fisher’s exact test, *P* = 0.0201), indicating that *sak* may be preferentially maintained in MRSA lineages. Among the 101 isolates, a total of 17 enterotoxin genes (*sea–seu*) were detected. MRSA strains carried a higher average number of enterotoxin genes per isolate (mean: 6.1) compared to MSSA strains (mean: 3.0). Genes from the enterotoxin gene cluster (*seg*, *sei*, *sem*, *sen*, *seo*) were predominantly found in MRSA (mean presence rate: 61.5%) and less so in MSSA (mean: 26.8%), suggesting that this cluster may be enriched in MRSA. Although *seb* was commonly detected in both groups, MRSA strains rarely carried *sed*, *seh*, *sej*, and *ser*. Interestingly, some MSSA isolates exclusively harbored *seh* (nine strains), *sed* (six strains), or *ser* (eight strains), pointing to the lineage-specific retention of these toxins. For the *tst* gene, 28.2% of MRSA (11/39) and 5.4% of MSSA (4/74) isolates were positive. The difference was statistically significant (Fisher’s exact test, *P* = 0.0037), highlighting a greater prevalence of the toxic shock syndrome toxin gene among MRSA strains.

Mobile genetic elements (MGEs), including insertion sequences, transposons, and prophages, displayed distinct distribution patterns among *S. aureus* isolates. MRSA strains more frequently harbored IS*Lgar5*, Tn*554*, and prophages, such as *Staphy phi2958PVL* and *PT1028*, which are closely associated with resistance and virulence genes. In contrast, MSSA isolates exhibited more diverse MGE profiles, including *ISSau3*, *ISSep3*, and *Staphy P282*. Notably, strains sharing similar MGE spectra often clustered together phylogenetically, suggesting co-acquisition events or clonal expansion. These findings underscore the role of MGEs in shaping the genomic plasticity and pathogenic potential of *S. aureus* lineages.

## DISCUSSION

MRSA remains as a significant public health threat in China (19), and understanding the molecular characteristics of *S. aureus*, especially MRSA, is crucial for tracking its evolutionary trends and implementing more effective prevention and control strategies.

Our analysis of 204 domestic *S. aureus* isolates reveals a complex molecular epidemiology in China, with both MRSA and MSSA strains showing frequent genetic exchanges and a lack of strict lineage segregation. This suggests a dynamic evolutionary process within *S. aureus*. Our findings confirm that CC59-ST59 is the predominant community-associated MRSA clone in China in line with previous reports from China and other parts of Asia (20). The broad geographic distribution of this clone, alongside its association with SCC*mec* IV and spa type t437, reflects its strong adaptation and transmission capacities in community settings.

ST239 MRSA has formed a powerful combination of resistance and virulence through the acquisition of mobile genetic elements and gene mutations, making it a major pathogen in hospital infections in China for a period of time. The novel *S. aureus* cell wall-anchored protein gene sasX carried on *φSPβ-like* prophages plays a crucial role in enhancing the bacterium’s colonization, immune evasion, and biofilm formation abilities (21–23). Studies have shown that *sasX* is closely associated with ST239 (23, 24). However, the detection rate of *sasX* in ST239 has varied across different studies in recent years (25), and it has also been found to spread from ST239 to other ST types (24). In our study, only one of the ST239 strains carried *sasX* (16.7%, 1/6), and no clear phylogenetic differentiation was observed between this strain and the other 5 ST239 strains, which may partly reflect the gradual weakening of ST239’s colonization advantage in China. Overall, our study observed a decline in the dominance of ST239 HA-MRSA in China, while ST59-CA-MRSA strains showed a concurrent rise. This shift is consistent with trends in recent years (13, 26), suggesting the growing presence and high diversity of CA-MRSA in hospital settings.

Our previous research showed that ST5 had long competed with ST239 (17) before ST59 gradually became the dominant clone in recent years (18). Despite this shift, ST5 remains as a key *S. aureus* lineage in Chinese hospitals. Historically dominant in countries like the U.S. and Japan, ST5 is now globally widespread. In line with our earlier findings on the global evolution of ST5, particularly in China and the U.S., all ST5-MRSA isolates in this study carried SCC*mec* II, representing 33.3% (3/10) of ST5 strains (17). These results suggest that the ST5 isolates likely originate from the Asian lineage.

Since its first description in the 1980s, CA-MRSA has seen a steady rise in incidence, marking a shift in MRSA epidemiology worldwide (27, 28). Typically carrying smaller SCC*mec* elements and often producing the cytotoxin PVL, CA-MRSA strains are particularly adaptable and capable of spreading more efficiently (4, 29). However, in our study, the overall PVL carriage rate among CA-MRSA isolates was relatively low (6.3%, 1/16). The single PVL-positive CC59 strain harbored the lukS-PV/lukF-PV genes carried by prophage φ7247PVL, consistent with previous reports that φ7247PVL is more commonly detected within the CC59 lineage (30). Compared with other PVL-negative CC59-ST59-SCC*mec* IV strains, this PVL-positive CC59-ST338-SCC*mec* V isolate was recovered from a patient with an intra-abdominal infection and exhibited fewer antimicrobial resistance genes. These findings may suggest that ST59 clones, particularly those lacking PVL and with broader resistance profiles, are better adapted for persistence and transmission in hospital settings (31).

Notably, CA-MRSA in hospitals is characterized by its high genetic diversity, with multiple clones emerging and circulating in different regions. This diversity in CA-MRSA highlights the dynamic nature of MRSA evolution and suggests that CA-MRSA strains are increasingly adapting to hospital environments. The clustering of ST59-CA-MRSA in the phylogenetic tree further suggests the potential outbreak or expansion of this lineage within healthcare settings. ST59 has become the dominant CA-MRSA clone in Asia, particularly in China, and is increasingly recognized for its enhanced pathogenicity. Previous studies have shown that ST59-t437-MRSA exhibits higher virulence than ST239-t030-MRSA, with greater toxicity and resistance to human blood (15). This increased virulence may contribute to higher mortality rates in patients with hospital-acquired *S. aureus* infections. Our previous research has also demonstrated that the *chp* gene enhances the virulence of ST59, further supporting the pathogenic potential of this clone (32).

Long-term exposure to hospital antibiotics may drive CA-MRSA to develop resistance to multiple drugs, complicating clinical management. The acquisition of mobile genetic elements (MGEs), such as plasmids, pathogenic islands, and transposons, plays a significant role in enhancing the virulence and resistance of MRSA strains (33). Our accessory genome analysis revealed that MRSA strains, particularly HA-MRSA, carry more enterotoxin genes and MGEs than MSSA, which may contribute to the higher virulence of MRSA.

While this study provides valuable insights into the emergence and diversification of CA-MRSA in Chinese hospitals, it is not without limitations. The relatively small sample size may not fully reflect the nationwide prevalence of *S. aureus*. In addition, the phylogenetic analysis was based on core genome alignment using Roary, which may have a lower resolution compared to SNP-based approaches derived from raw read mapping. This could underestimate fine-scale evolutionary differences among closely related strains. Nevertheless, our findings underscore the importance of ongoing surveillance to monitor the evolving landscape of MRSA in China and its potential clinical implications.

### Conclusion

Our study revealed the high genetic diversity and the widespread dissemination of *S. aureus* in Chinese hospitals, particularly CA-MRSA. Our findings suggest that CA-MRSA represented by the ST59 clone is gradually replacing traditional HA-MRSA lineages and emerging as a major cause of hospital infections, underscoring the need for enhanced molecular surveillance and targeted infection control strategies.

## SUPPLEMENTAL MATERIAL

Supplemental material spectrum.03395-24-s0001.docx Fig. S1 and S2.

Table S1 spectrum.03395-24-s0002.xlsx Cities, infections, and molecular types of isolates.

Table S2 spectrum.03395-24-s0003.xlsx MIC of antibiotics.

Table S3 spectrum.03395-24-s0004.xlsx Acquired antibiotic resistance genes.

Table S4 spectrum.03395-24-s0005.xlsx Virulence genes.

Table S5 spectrum.03395-24-s0006.xlsx MGEs.

ASM does not own the copyrights to Supplemental Material that may be linked to, or accessed through, an article. The authors have granted ASM a non-exclusive, world-wide license to publish the Supplemental Material files. Please contact the corresponding author directly for reuse.
