# Supplementary material for: Unveiling the emergence of community-acquired methicillin-resistant Staphylococcus aureus (CA-MRSA) in Chinese hospitals with remarkable genetic diversity: a multicenter molecular epidemiological investigation
Source: Microbiol Spectr. 2025 Oct 30;13(12):e03395-24. doi: 10.1128/spectrum.03395-24 (PMC12671212; doi:10.1128/spectrum.03395-24)
Supplement: Supplemental material — Fig. S1 and S2. [file spectrum.03395-24-s0001.docx]

Supplement materials


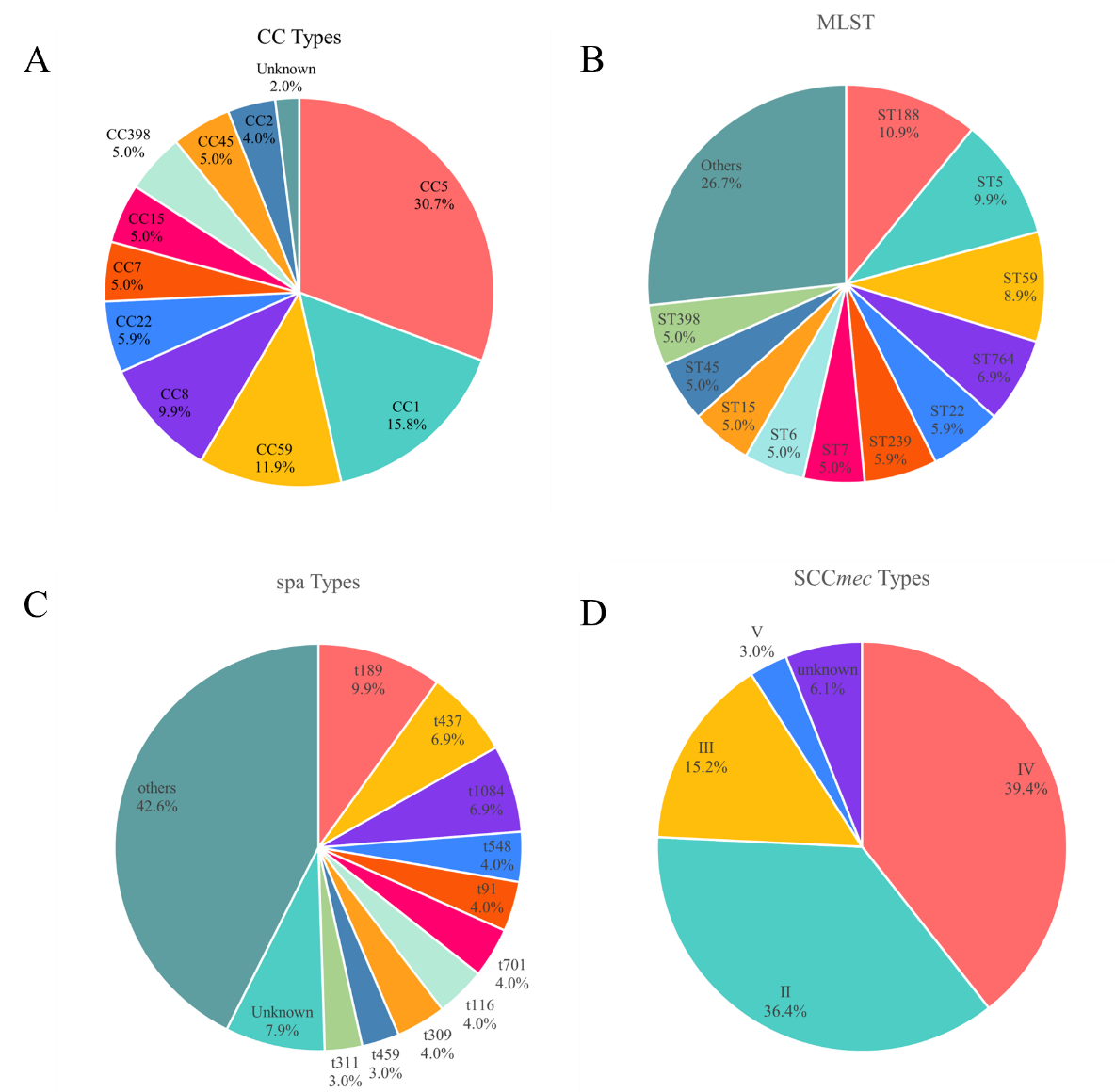


**Fig S1**. Molecular typing distribution of the *S.aureus*. (A) Numbers and proportions of *S.aureus* of different ST types. (B) Numbers and proportions of *S.aureus* of different spa types. (C) Numbers and proportions of *S.aureus* of different CC types. (D) Numbers and proportions of MRSA of different SCC*mec* types.


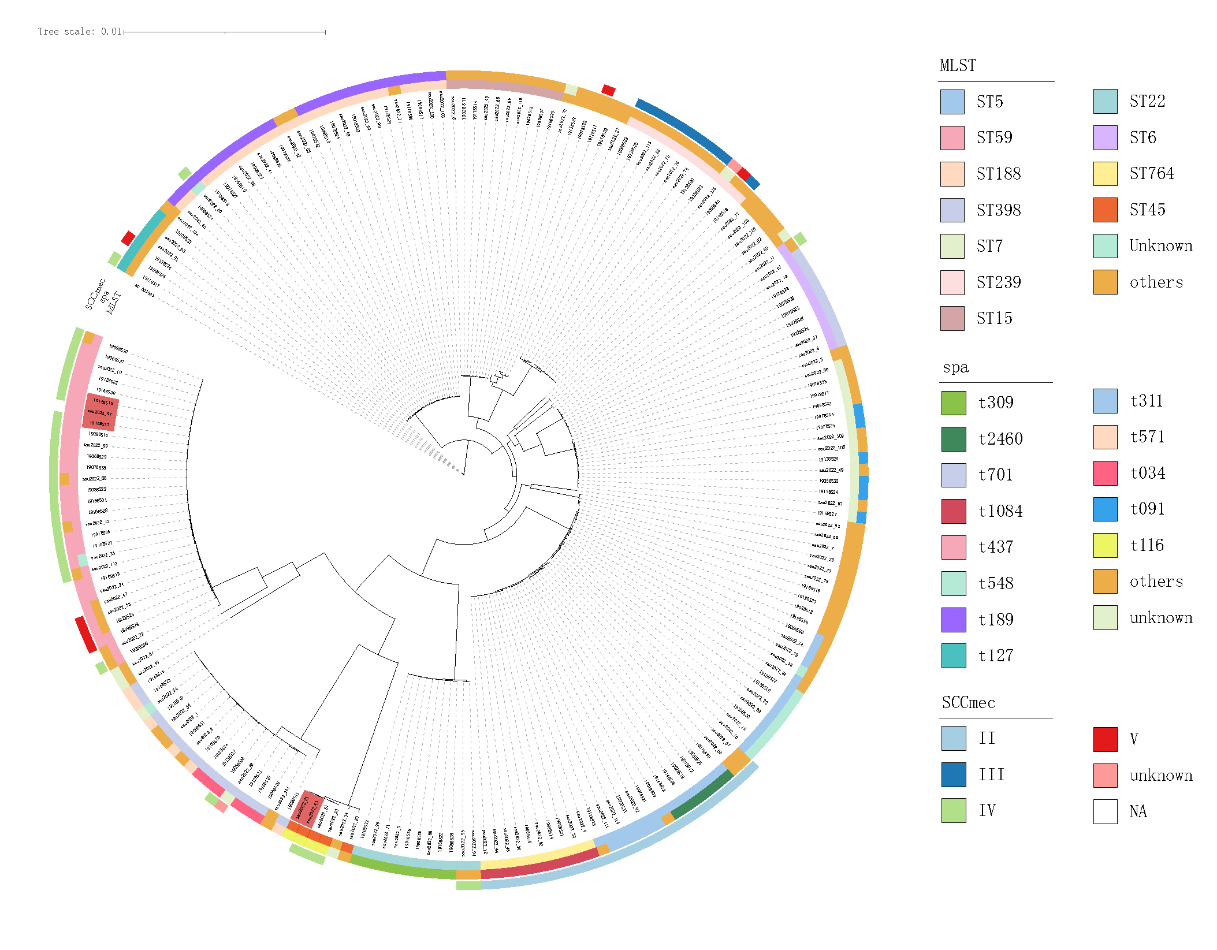


**Fig S2**. Core genome-based phylogenetic tree of Staphylococcus aureus isolates (n = 204). From the innermost to the outermost ring, the annotated metadata layers represent MLST, *spa* type, and SCC*mec* type, respectively. Metadata colors indicate different allele types or subtypes as defined in the legend. Strains with identical sequence types but differing methicillin resistance profiles (MRSA vs. MSSA) and clustering within the same phylogenetic branch are marked in red.
